# Biochemical characterization of Soxhlet-extracted pulp oil of *Canarium schweinfurthii* Engl. fruit in Nigeria

**DOI:** 10.1038/s41598-022-14381-w

**Published:** 2022-06-18

**Authors:** Kingsley O. Omeje, Benjamin O. Ezema, Juliet N. Ozioko, Henry C. Omeje, Emmanuel C. Ossai, Sabinus O. O. Eze, Charles Odilichukwu R. Okpala, Małgorzata Korzeniowska

**Affiliations:** 1 Department of Biochemistry, University of Nigeria, Nsukka, Enugu Nigeria; 2 Department of Biochemistry, University of Port Harcourt, Port Harcourt, Rivers State Nigeria; 3 Department of Functional Foods Product Development, Wrocław University of Environmental and Life Sciences, 51-630 Wrocław, Poland

**Keywords:** Biochemistry, Biotechnology

## Abstract

Characterization and further development of underutilized/underexploited indigenous tropical seed oils are essential to supplement both nutritional and industrial needs of an ever-increasing African (and global) population. Before now and to our best knowledge, the previous research involved *Canarium schweinfurthii* Engl. fruit specific to Nigeria appear to have been more on the evaluation of seed, pulp, and essential oils (from the seed), but much less on the pulp oil. To supplement existing information, this current work has aimed to biochemically characterize the Soxhlet-extracted pulp oil of *C. schweinfurthii* fruit gathered from a community situated in the South-east of Nigeria. Specifically, the biochemical characterization comprised the determinations of proximate compositions, lipid peroxidation, fatty acid profile, as well as carotenoids, sterols, and tocopherols. Processing the fruit sample to pulp oil involved, among others, oven-drying, and grinding, prior to the Soxhlet extraction. Results of proximate components of *C. schweinfurthii* pulp oil showed the following trend: crude fat content (~ 49.32%) > carbohydrates (~ 37.93%) > moisture content (~ 8.62%) > ash content (~ 3.74%) > crude protein content (~ 0.39%) values. The lipid peroxidation attributes comprised acid (~ 23.60 mg KOH/g), peroxide (~ 33.91 mEq. O_2_/kg), iodine (~ 58.3 g/100 g), and saponification (~ 138.21 mg KOH/g) values. In addition to the free (~ 13.8%), saturated (~ 9.74%), and unsaturated (~ 90.26%) fatty acids, a total of fifteen (15) fatty acid methyl esters (FAMEs) spectral peaks were found, from caprylic acid (C8:0) to lignoceric acid (C24:0). Total tocopherol concentration amounted to ~ 73 mg/100 g, which comprised α, β, γ-tocopherol, and δ-tocotrienol, with fair concentrations of carotenoids and sterols. Overall, the *C. schweinfurthii* pulp oil—biochemically competitive with a high concentration of unsaturated fatty acid, tocopherol, and sterol, suggests strong industrial promise.

## Introduction

Globally, many plant foods still avail themselves as very useful essential oil candidates—some are still underutilized, whereas others are increasingly utilized^1^. Further arising from global population increases, the development of already existing yet underutilized crops should help avert the prevailing food crises, which could help improve the developing economies, and contribute as industrial raw materials. In the recent decade, the research interest to harness underutilized food crops that targets enhanced nutritional and industrial applications is on the rise particularly in Africa. Transforming underutilized wild fruits/oilseeds into alternative nutritional value would be augmenting the inadequate supplies of animal sources^2^. Specifically*, Canarium schweinfurthii* Engl. is among the underutilized crops that increasingly thrive across the tropical Africa rain and transitional forests across countries like Cameroon, Congo, Cote d’Ivoire, Gabon, Senegal, extending to other countries like Angola, Ethiopia, and Tanzania^3–6^. Characterized by cylindrically straight bole with the crown that nears the upper canopy, the tree provides promising shade cover with resultant wood for timber^4,7–10^. The *C. schweinfurthii* tree in Nigeria is given local names like African elemi (English)*, Atilis* (Hausa), *Ube agba* (Igbo) and *Elemi* or *Agbabubu* (Yoruba), and purple canary tree^11^. Besides producing fruits largely between the April and September months, the *C. schweinfurthii* tree possesses flowers that clusters at the twig end^3,6,12^. Barks of the *C. schweinfurthii* tree serve as resource for ointment, plaster and printing ink preparations. A cut on the bark of the tree would exude the gum that eventually solidifies into a whitish resin^7^. Further, the fruit may appear olive-like, long spiral, short ovoid-shaped, having single triangular seeds with tiny-like projections at the three edges^3,4,12^. Moreover, when ripe the fruit appear purplish in the forest, but dark brown in the savannah regions^5^. Edible fleshy pulp of *C.* *schweinfurthii* fruit is regularly boiled and retailed at open food markets^3,11^.

The characteristic potentials of *C. schweinfurthii* fruit, especially across the African continent, appears to be of increased research interest. Nagawa, Böhmdorfer, and Rosenau^13^ studied the chemical composition and anti-hermitic activity of *C. schweinfurthii* essential oil obtained from Sango Bay Area, Southern Uganda where the essential oil’s main constituents were reported as monoterpenes. Edou et al.^14^ studied the volatile constituents of *C. schweinfurthii* essential oil obtained from Gabon, and found limonene, sabinene, α-pinene as the major components that constituted ~ 81.90% of the essential oil, with the monoterpenoids predominant of the terpenoid components. Koudou et al.^15^ reported the chemical composition and pharmacological activity of *C. schweinfurthii* essential oil obtained from the Central African Republic, where the major constituents included octylacetate (60%) and nerolidol (14%). Dongmo et al.^2^ reported the chemical characterization, antiradical, antioxidant, and anti-inflammatory potential of essential oils from the *C. schweinfurthii* plant in Cameroon. Major compounds found included p-cymene, limonene and α-terpineol in varied quantities. Specific to Nigeria, Maduelosi and Angaye^8^ characterized the *C. schweinfurthii* fruit seed and pulp obtained from Ebonyi State Nigeria, specific to some physicochemical attributes. Atawodi^16^ studied the polyphenol composition and in vitro antioxidant potential *C. schweinfurthii* fruit obtained from Plateau State, Nigeria. Abayeh, Abdulrazaq and Olaogun^3^ investigated the bound/flowing lipid contents of mature *C. schweinfurthii* fruit endocarp/mesocarp. However, Georges, Olivier, and Simard^4^ seems to be the only one that had examined the physicochemical composition of *C. schweinfurthii* pulp oil from the fruit harvested at Cote d’Ivoire, wherein the obtained results were compared with other vegetable oils.

Improving and optimizing the existing extraction processes, from laboratory to industrial scale that employs low environmental impact, continues to pose a great challenge, particularly as the globe strives to go green^17,18^. Additionally, developing/delivering a green extraction laboratory, particularly on an industrial scale comes with great challenges. Discussing the raw material consumption and optimal energy involving solvents, Ivanovs and Blumberga^19^ considered green extraction methods like enzymatic hydrolysis, microwave-assisted extraction (MAE), supercritical fluid extraction using CO_2_ (SCF-CO_2_), and ultrasound-assisted extraction (UAE). However, these extraction methods remain costly and not easily afforded by all laboratories, especially in developing countries. More so, the rising demand for food oils for human consumption/industrial applications makes many parts of the globe still dependent on low-cost extraction processes^6,20^. That is why the agro-food industry as well as researchers particularly in the developing countries, in order to achieve high yields of bioactive compounds from food materials, still utilize the conventional/solvent extraction protocols where the Soxhlet method is among them^21^. What makes the Soxhlet method stand unique is its rugged and well-established process, as well as the rather low-cost affordable and unattended extraction process. More so, the operation of Soxhlet method is relatively simple and provides promising/reliable results^6,21^, despite the demerits like the evaporation required at the end of the extraction, large solvent volumes, as well as long operation time (several hours), all of which continues to drive the chemical/food industries to continue to search for the environmental-friendly extraction methods^22^.

The extraction of essential oils from raw plant materials frequently employs the Soxhlet method. The latter particularly in developing countries is still the standard extraction method of choice across many chemical laboratories/industries^22^. Moreover, if the bioactive components of underutilized perennial food crops like the *C.* *schweinfurthii* fruit available in many communities in Africa are to be harnessed, then, more sustainable approaches would surely be needed. Before now, researches involving *C. schweinfurthii* fruit have focused more on evaluation of its seed, pulp, and essential oils from the seed. More importantly, there is a paucity of relevant literature about the pulp oil extracted from the *C. schweinfurthii* fruit, particularly those cultivated in Nigeria, and to our best knowledge. To supplement existing information, this current work has aimed to biochemically characterize the Soxhlet-extracted pulp oil of the *C. schweinfurthii* fruit gathered from a community situated in South-east of Nigeria. Investigating the biochemical properties—proximate compositions, lipid peroxidation, fatty acid profile, determinations of carotenoids, sterols and tocopherols—of the Soxhlet-extracted pulp oil would provide important insights into the nutritional and industrial applications (of the oil). More so, such additional information would help consolidate the product development potential(s) of this underutilized oil crop/plant.

## Materials and methods

### Schematic overview of the experimental program

**Figure 1:**
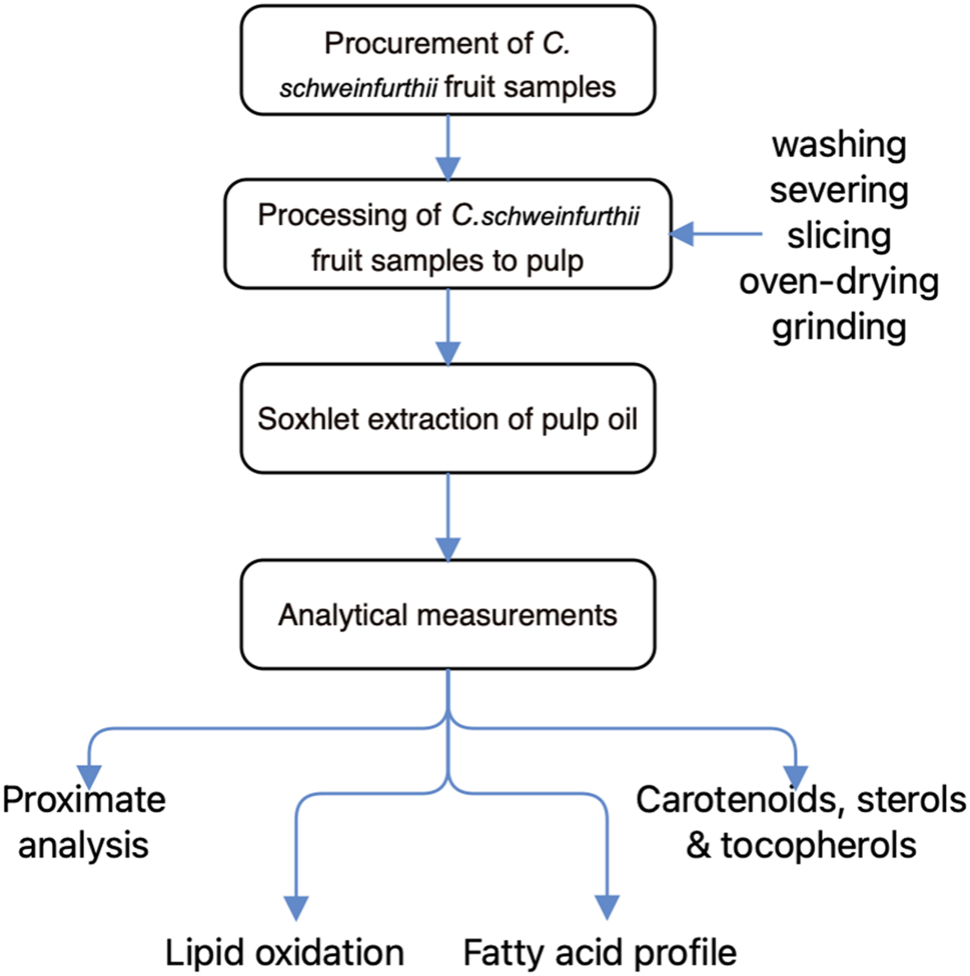
A schematic overview of the experimental program of this current study, showing the major stages, from procurement of *C. schweinfurthii* fruit samples, processing into the pulp, followed by the Soxhlet extraction of its oil, and subsequent analytical measurements.

The schematic overview of the experimental program of this current study, which depicts the major stages from procurement of fruit samples, processing into the pulp and followed by the Soxhlet extraction of its oil, prior to the subsequent analytical measurements, is shown in Fig. 1. For emphasis, this conducted research was directed to provide additional information about the biochemical promise of the pulp oil of *C. schweinfurthii* fruit cultivated in Nigeria. Specifically, the biochemical characterization comprised the determinations of proximate compositions, lipid peroxidation, fatty acid profile, as well as carotenoids, sterols, and tocopherols. Additionally, the analytical measurements were performed independently using different pulp oil samples obtained from one batch of *C. schweinfurthii* fruit. Importantly, all the conducted analytical measurements adhered to the relevant guidelines set out by the Department of Biochemistry, University of Nigeria, Nsukka, Enugu State, Nigeria.

### Chemicals and reagents

N-hexane, potassium iodide (KI), sulphuric acid, boric acid, sodium sulfate, diethyl ether, potassium hydroxide, sodium thiosulphate, acetone, acetic acid, chloroform, ethanol, and methanol were procured from Guangdong Chemical Factory (Guangdong, China). Ethylacetate, Wijjs reagent, isopropyl alcohol (lichrosolv), alpha/gamma tocopherol, ergosterol, cholecalciferol, ergocalciferol, campesterol, and sitosterol were procured from Sigma-Aldrich (USA). Others like tetrahydrofuran (THF), acetonitrile, betacarotene, alpha/gamma carotene reference standards were procured from Shandong Yanshuo Chemical Co., Ltd. (Linzi, China). All chemicals/reagents employed in the study were of analytical grade standard.

### Collection, preparation, and processing of *C. schweinfurthii* fruit to pulp

The freshly (mature fruit) harvested samples, obtained from various wild *C. schweinfurthii* trees and gathered as one batch (~ 90 g), were picked from Edem-ani community (6°51′43″N 7°20′21″E) of Nsukka local government area (LGA), Enugu State, South-east of Nigeria. Permission to collect the fruit samples was given by the farmers that owned the various plant fields, which availed the wild *C. schweinfurthii* trees. In addition to the collection process that adhered to the prescribed plant material guidelines, the taxonomy identification of *C. schweinfurthii* fruit samples was performed by Mr. Felix Okoli (plant taxonomist) at the Plant Science and Biotechnology Unit, University of Nigeria Nsukka, and voucher specimen has been deposited in the herbarium for reference purposes (Voucher reference number = PCG/UNN/0407*Canarium schweinfurthii* Engl [Burseraceae]). From the assembled batch, the fruit samples were randomly selected, and seeds separated following the method described by Abayeh, Abdulrazaq and Olaogun^3^ with modifications, to secure the succulent fruit pulp (cotyledon). This involved washing the fruit pulp, severing it from the hard nut, slicing, and thereafter, subject to oven drying at ~ 50 °C for 8 h, before grinding using the electric blender. Subsequently, the ground *C. schweinfurthii* pulp was then ready for the Soxhlet extraction.

### Soxhlet extraction of *C. schweinfurthii* pulp oil

To produce the *C. schweinfurthii* pulp oil, the Soxhlet extraction method using n-hexane as the solvent, previously described in the AOAC method^23^ with some modifications, was employed. This involved weighed *C. schweinfurthii* ground fruit pulp sample (~ 15 g) with ~ 150 mL of n-hexane solvent submitted to Soxhlet extractor that operated at temperature of ~ 65 °C. The extraction period lasted for about 4 h. When the extraction had completed, the residual solvent was allowed to evaporate, and the free oil was quantified as yield, and recorded by percentage. The pulp oil was recovered, and preserved in a sample bottle, and stored at 4 °C until required for further analysis.

### Analytical measurements of *C. schweinfurthii* pulp oil

#### Proximate analysis

Proximate analysis involved the determinations of crude protein, crude fat, moisture, ash, and carbohydrate contents, as well as nitrogen free extract using the AOAC method^24^ with some modifications.

To determine the crude protein content, fresh oil sample (~ 0.3 g) was weighed into a Kjeldahl flask with 0.20 g catalyst. The digestion used ~ 10 mL concentrated H_2_SO_4_, 50 mL of 4% boric acid, followed by three drops of methyl red. Thereafter, 40% NaOH (25 mL) was added, after which the distillate was titrated against 0.5 N Na_2_SO_4_. With % N available, the determination of crude protein was established using the correction factor (% N × 6.25).

To determine the crude fat content, fresh oil sample (~ 0.3 g) was weighed into an extraction thimble and placed into a quick fit Soxhlet apparatus (Merck KGaA, Darmstadt, Germany) with solvent flask containing 25 mL of diethyl ether connected to a condenser. The extraction completed in ~ 6 h, and extract was evaporated at ~ 70 °C to remove any remaining solvent present. The apparatus was reweighed, and percentage fat was calculated as follows:

$$\mathrm{\% Fat }= \frac{Weight \; of \; oil}{Weight\;of\;sample} \times 100$$

To determine the moisture content, fresh oil sample (~ 0.5 g) was weighed and dried in the oven at 110 °C to a constant weight. The dish and sample were cooled and reweighed and percentage moisture content was determined and expressed as percentage.

To determine the ash content, previously weighed porcelain dishes had fresh oil sample (~ 3 g) subject to muffle furnace at 600 °C for ~ 3 h. The percentage ash content were calculated using the equation below:

$$\mathrm{\% Ash }=\frac{W_{3}-W_{1}}{W_{2}-W_{1}}$$

where: W_1_ = weight of crucible; W_2_ = Weight of crucible and sample; W_3_ = Weight of crucible and ash.

In order to determine the nitrogen free extract (NFE), the crude fibre content had to be determined first, using fresh oil sample (~ 3 g), which had been subjected to diluted H_2_SO_4_, boiled for 30 min and filtered. Subsequently, ~ 150 mL of pre-heated KOH and drops of octanol were added, followed by boiling for ~ 30 min, and thereafter filtered. Thereafter, acetone was used to wash the sample for three times in the cold extraction unit, after which the content was dried at 130 °C for 1 h, and then ashed at 500 °C. The ash was weighed and percentage crude fibre calculated using the equation below:

$${\% \text{Crude} \; \text{fibre} }= \frac{Weight \; of\; fibre}{Weight\;of\;sample} \times 100$$

With this crude fibre information now available, the %NFE was determined by subtracting the sum of other fraction from 100 as follows: 100 − (% moisture + % protein + fat + fibre + ash) = % NFE. Furthermore, the total carbohydrate content was elucidated after all other components have been measured.

#### Lipid peroxidation analysis

Regarding the acid value, the AOAC method^24^ with slight modifications was applied to the *C. schweinfurtaii* pulp oil. This involved the use of the mixture of ethanol and diethyl ether, 25 mL (denatured alcohol) (v/v), then 3 drops of phenolphthalein indicator neutralized with 0.1 M ethanolic KOH solution. About 0.5 mL of the oil samples were added to the neutralized solution, and finally titrated against 0.1 M ethanolic KOH solution, to reach permanent pink colour. Expressed as mg KOH/g, the acid value (A.V.) was calculated as follows:

$$\mathrm{A}.\mathrm{V}.=\frac{Vol.\;of\;KOH\;used \times Mass\;of\;KOH}{Mass\;of\;sample}$$

Regarding the peroxide value, the AOAC method^24^ with slight modifications was applied to the *C. schweinfurthii* pulp oil. First, the oil (0.5 mL) was dissolved in a solvent mixture of acetic acid and chloroform (1:2). Then, KI (~ 1.3 g) was added, and mixture placed in a dark cupboard for 1 h, after which ~ 75 mL of distilled water was added, followed by 3 drops of starch indicator, and titrated against 0.05 M sodium thiosulphate. The peroxide value, expressed as millimoles of active oxygen per kilogram (mEq. O_2_/kg), was calculated as follows:

$$\mathrm{P}.\mathrm{V}.=\frac{S \times N \times 1000}{weight\;of\;sample}$$

S = (Vol. of Na_2_S_2_O_3_ for blank – Vol. of Na_2_S_2_O_3_ for sample), N = Normality of Na_2_S_2_O_3_.

Regarding the iodine value, the Wijs method as described by Firestone^25^ with slight modifications was applied to the *C. schweinfurthii* pulp oil. The pulp oil samples (~ 0.5 g) has been mixed with chloroform (~ 5 mL) and Wijjs reagent (~ 8 mL), (which comprised ~ 9 mL of iodine trichloride and 10 g of iodine in chloroform (~ 300 mL)/acetic (~ 700 mL) solution), swirled and placed in the dark cupboard for 1 h after which ~ 7 mL of KI and ~ 35 mL of distilled water were added, and titrated against 0.05 M Na_2_S_2_O_3_·5H_2_O solution using starch as the indicator. A blank test was carried out simultaneously using water in place of the oil under the same conditions. Expressed as g/100 g, the iodine value (I.V.) was calculated as follows:

$$\mathrm{I}.\mathrm{V}.=\frac{\left(Blank{\text{-}}sample\right) \times 0.01269}{W} \times 100$$

Regarding the saponification value, the indicator method as described by Lamani et al.^20^ with slight modifications was applied to the *C. schweinfurthii* pulp oil. The alcoholic KOH solution was refluxed, with pulp oil sample (~ 0.5 g). Thereafter, ~ 30 mL of 0.1 M of ethanolic KOH has been added, and allowed to boil for ~ 30 min under the reflux. Few drops of phenolphthalein indicator were added, followed by titration against 0.5 M HCl until the disappearance of the pink colour (end point). A similar procedure was administered to achieve the blank. Expressed as mg KOH/g, the saponification value (S.V.) was calculated as follows:

$$\mathrm{S}.\mathrm{V}.=\frac{56.1\left(Blank{\text{-}}sample\right) \times N}{W}$$

where N = Actual normality of the HCl used; W = mass of the pulp oil sample.

#### Fatty acid profile analysis

The method previously described by Aremu, Ogunlade, and Olonisakin^26^ with modifications was employed to determine the fatty acid profile of the *C. schweinfurthii* pulp oil. The process was conducted using Perkin-Elmer Clarus 500 gas chromatograph (GC) (Billerica, MA), equipped with a flame-ionization detector (FID). The column type was Hewlett-Packard INNOWax (Hewlett-Packard Co, Palo Alto, CA) with a dimension of 30 m × 0.25 mm × 0.25 μm. The fatty acids methyl esters (FAMEs) mixture (AccuStantard Inc., New Haven, CT) served as the reference external standard, while margaric acid methyl ester (C17:0) was used as the internal standard. The external fatty acids methyl esters mixture standard comprises C8:0, C10:0, C12:0, C14:0, C16:0, C16:1, C18:0, C18:1, C18:2, C18:3, C20:0, C20:4, C22:0, C22:1, and C24:0. The *C. schweinfurthii* pulp oil sample (~ 0.5 g) was saponified (esterified) for 5 min at 95 °C, with ~ 3.4 mL of KOH (0.5 M) in dry methanol. The mixture was neutralized using HCl (0.7 M) and ~ 3 mL of boron triflouride (14%) in methanol. After, the mixture was heated for ~ 5 min at 90 °C to achieve complete methylation process. The fatty acids methyl esters were extracted from the mixture with redistilled n-hexane, and then concentrated to ~ 1 mL for further analysis. The fatty acid methyl ester composition of the sample was analyzed by the injection of 1 μL of sample. The carrier gas was nitrogen with a split ratio of 1:20. The injector and detector temperatures were respectively 250 and 320 °C. The first ramping was at 12 °C/min, which lasted for ~ 20 min, and maintained for ~ 2 min. The second ramping was at 15 °C/min, which lasted for ~ 3 min, and maintained for ~ 8 min. The peaks of the fatty acid methyl esters were verified based on retention times with those of external standard (AccuStantard).

#### Determination of carotenoids, sterols and tocopherols

The method described by Czaplicki, Tańska, and Konopka^27^ with modifications was employed for the determinations of carotenoids, sterols, and tocopherols in *C. schweinfurthii* pulp oil, which employed high-performance liquid chromatography (HPLC) instrument (Hangzhou-LC-8518, Zhejiang, China). Specifically, the HPLC ultraviolet (UV) detector supported by N200 chromatography software helped to establish the chemical constituents of carotenoids, sterols, and tocopherols contents. The HPLC instrument operated with a low-pressure gradient, solvent delivery pump, high-pressure switching valve, as well as high-sensitivity UV detector. Column size was 150 × 4.6 mm, with an injected sample volume of ~ 40 mL. Mobile phase was set for carotenoids (Acetonitrile/Methanol/Water/THF, 70:20:8:2), tocopherol, and sterols (n-hexane/ethylacetate, 70:30), using wavelength (Lamda maximum) of 450 nm, column temperature of ~ 40 ºC, and run time of ~ 20 min. Results of carotenoids, sterols, and tocopherols were expressed as µg/100 ml.

### Statistical analysis

All data from duplicate measurements of different pulp oil samples of one *C. schweinfurthii* fruit batch, were subject to a simple t-test. Where applicable, the results were presented in terms of means ± standard deviations (SD). Statistical Package for the Social Sciences (SPSS) Software version 16 (SPSS Inc., Chicago, Illinois, USA) was used to run the data.

## Results and discussion

### Proximate and yield analysis of *C. schweinfurthii* pulp oil

The proximate components of the *C. schweinfurthii* pulp oil were determined, as shown in Table 1, which showed the following trend: crude fat content (49.32 ± 0.07%) > carbohydrates (37.93 ± 1.70%) > moisture content (8.42 ± 1.05%) > ash content (3.74 ± 0.23%) > crude protein content (0.39 ± 3.41%) values. These proximate differences could depend on factors like geographical location, as well as harvest season. Georges, Olivier, and Simard^4^ reported *C. schweinfurthii* fruit pulp from Cote d’Ivoire with 5.6% protein, 30–50% fat, 8.2% starch, as well as 8.3% ash contents. Agu, Ukonze, and Uchola^12^ reported the crude fat and moisture content of *Atili* oil (crude fat content = 22.82%, moisture content = 8.62%), which appeared higher than those of the *C. schweinfurthii* pulp oil at this current study. Probably, the steps involved in the processing the fruit into pulp might have contributed to lessen the crude fat and protein and increased carbohydrate contents of the *C.* *schweinfurthii* pulp oil. Other factors such as location, cultivation practices, age of the fruits may contribute to the observed proximate differences. Elsewhere, Nyam et al.^9^ reported *C. schweinfurthii* fruit samples with proximate values of 64.04% crude fat, 6.39% protein, 16.37% fibre, and 3.85% carbohydrate.

**Table 1:** Proximate components of *C. schweinfurthii* pulp oil.

Proximate components	Quantity (%)
Ash	3.74 ± 0.23
Moisture	8.42 ± 1.05
Crude fat	49.32 ± 0.07
Crude protein	0.39 ± 3.41
Carbohydrates	37.93 ± 1.70

The *C. schweinfurthii* pulp oil was successfully extracted using the Soxhlet extraction technique that employed n-hexane as the solvent and operated at 70 °C for 4 h. In particular, the oil yield of *C. schweinfurthii* fruit was ~ 53.69%, which somewhat resembled those data reported by Nagawa, Böhmdorfer, and Rosenau^13^, but above those data reported by Dongmo et al.^2^. Possibly, among other factors, the moisture content in the *C. schweinfurthii* fruit sample may have influenced the pulp oil yield of this current study. Additionally, the extent of the high oil yield may well be associated with the part of plant used. Besides, Dongmo et al.^2^ understood that differences in oil yield from *C. schweinfurthii* fruit might depend on the place of harvest, and this is what Ndoye^28^ observed when investigating *C. schweinfurthii* fruit resins from the East Region of Cameroon, where the Ebouete, Lomie and Mbeth species/varieties respectively recorded 8.6%, 7.6%, and 9.3% yield. Other plant seed oil yields reported lower values compared to those found in this current work, for instance, *Chrysophyllum albidum* varieties (oil yield range = 3.52–3.75%)^29^, *Persea americana* seed (oil yield = 36.93%)^30^, African star cherry (oil yield = 23.80%)^31^, seed oil of *Lophira lanceolata* (oil content = 40.0%) and *Schoro caryabirrea* (oil content = 42.0%)^32^. Therefore, the ~ 53.69% oil yield obtained for *C. schweinfurthii* fruit at this study makes it a promising oil resource for both industrial and nutritional purposes compared to other underutilized plants/crops.

### Lipid peroxidation of *C. schweinfurthii* pulp oil

Among very important quality criteria in the food industry is the lipid breakdown levels of plant/seed oil. This is largely because the (lipid oxidation) process produces rancid flavours that decrease the food product’s nutritional quality/safety. Also called auto-oxidation, this process can be quite complex especially across edible oils given its dependency on conditions of oxidation, and oil types^33^. The lipid and fatty acid contents of *C. schweinfurthii* pulp oil are shown in Table 2. Besides being of a pleasant odour with dark green colour and liquid at 28 °C, the pulp oil comprised acid (23.60 ± 2.35 mg KOH/g), iodine (58.3 ± 0.57 g/100 g), peroxide (33.91 ± 0.80 mEq. O_2_/kg), and saponification (138.21 ± 2.04 mg KOH/g) values, along with some quantities of free (13.8%), saturated (18.97%), and unsaturated (80.97%) fatty acids. Specifically, our iodine and peroxide values clearly differ from those data reported by Georges, Olivier, and Simard ^4^ for *C. schweinfurthii* pulp oil. Essentially, the acid value demonstrates, not only freshness of the pulp oil and its constituent free fatty acids, but also, the degree at which the triglycerides therein has been hydrolyzed by the lipase^29,34^. A low acid value suggests reduced degree of hydrolytic and lipolytic activities in the oil sample^32^. Of the current work, the acid value of *C. schweinfurthii* pulp oil (23.60 ± 2.35 mg KOH/g) appeared above those of African star apple (13.60 ± 2.35 mg KOH/g)^31^. Other workers like Agu, Ukonze, and Uchola^12^ reported acid value and free fatty acid content of 0.62 mg KOH/g and 1.98% for *Atilis* oil, whereas Omeje, Ozioko, and Omeje^30^ reported acid value and free fatty acid content of 7.86 mg KOH/g and 8.75% for *P. americana* seed oil, respectively.

**Table 2:** Lipid and fatty acid contents of the *C. schweinfurthii* pulp oil.

Lipid and fatty acid contents	Oil sample
Odour	Pleasant
Colour	Dark green
State at 28 °C	Liquid
Acid value	23.60 ± 2.35 mg KOH/g
Peroxide value	33.91 ± 0.80 mEq. O_2_/kg
Iodine value	58.3 ± 0.57 g/100 g
Saponification value	138.21 ± 2.04 mg KOH/g
Free fatty acid content	13.80%
Saturated fatty acid content	18.97%
Unsaturated fatty acid content	80.97%

Compared to iodine value (58.3 ± 0.57 g/100 g) of *C. schweinfurthii* pulp oil, there are other plant seed oils locally available within the same study area of this current work that have shown lower (iodine) values, for example, African star cherry seed oil (29.00 g/100 g)^31^ and *P. americana* seed oil (33.21 g/100 g)^30^. A peak iodine value of an obtained oil would signal a high degree of unsaturation^35^, which if put in the context of this current study, would suggest the relatively high unsaturated fatty acids of the *C. schweinfurthii* pulp oil (80.97%). Notably, peroxide values serves as initial oxidation products and relatively short-lived aspects of unsaturated fatty acids^34^. Further, peroxide values increase with the levels of oxidative rancidity and decrease with the levels of antioxidants^36^. Peroxide value of *C. schweinfurthii* pulp oil (33.91 ± 0.80 mEq. O_2_/kg) at this current study fell below that of Avocado seed oil (~ 42.11 mEq. O_2_/kg)^30^. Essentially, the desirable quality edible oils that enhance the storage time with little-to-zero deterioration are those associated with low peroxide/high iodine values^35^. Nonetheless, the processing of *C. schweinfurthii* cotyledon/fruit into pulp, alongside Soxhlet extraction applied to extract the oil, may contribute to influence the lipid oxidation outcomes at this current work. This could be considered given that Abayeh, Abdulrazaq, and Olaogun^3^ demonstrated different ranges in lipid oxidation of endocarp/mesocarp oil extracts specific to saponification value (SV) (endocarp = 95.4–184.3 mg KOH/g; mesocarp = 151.9–195.3 mg KOH/g), peroxide value (PV) (endocarp = 4.0–8.0 mEq. O_2_/kg; mesocarp = 20–40 mEq. O_2_/kg), iodine value (IV) (endocarp = 100.1–118.3 g iodine/100 g; mesocarp = 71.1–94.9 g iodine/100 g) and acid value (AV) (endocarp = 0.48–8.70 mg KOH; mesocarp = 1.33–8.30 mg KOH).

### Fatty acid profile of *C. schweinfurthii* pulp oil

Fatty acid profile, whether from animal or vegetable oils, together with their derivatives, such as alkyl esters, remain among strong influences on product’s chemical and physical properties. From the industrial and physiological standpoint, fatty acid profile shows the different numbers of double bonds in their aliphatic chain at different positions^37^. Additionally, gas chromatography (GC) remains among the widely used methods in determining the fatty acid composition/profiles, particularly for animal fats and vegetable oils, together with their derivatives. For this purpose, the oils or fats are typically converted to their corresponding methyl esters^22,37^. In the modern analytical chemistry, most GC instruments operate with cross-detector analysis, incorporating the flame ionization detector (FID). Typically, besides helping in delivering a wide range of organic compounds, the FID provides a resistance to small fluctuations especially to the gas flow, and insensitive to the arising gas impurities. More so, the response of FID appears very predictable, as it adheres to the rule of equal carbon response, and capably provides a lower relative standard deviation particularly for inter- and intra-reproducibility^38^.

**Figure 2:**
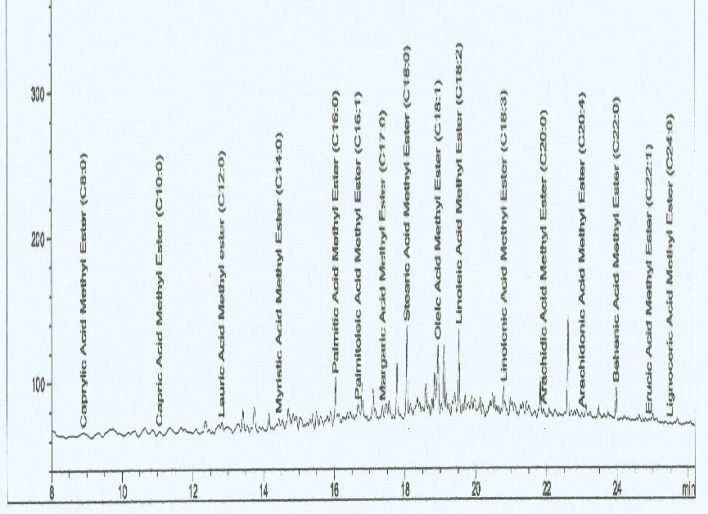
GC-FID chromatogram of *C.schweinfurthii* pulp oil.

The GC-FID chromatogram of *C. schweinfurthii* pulp oil is displayed in Fig. 2. In total, there were fifteen (15) FAMEs in the external standard used, directly reflective of the observed spectral peaks, given that margaric acid methyl ester (C17:0) served as an internal standard. Importantly, the percent of saturated and unsaturated fatty acids would be estimated based on the components of the FAMEs external standard used. To further elaborate on the peaks, the fatty acid profile of *C. schweinfurthii* pulp oil, based on compounds, retention time, concentration, carbon chain ratio, and chemical formulae is shown in Table 3. The fatty acid profile of *C. schweinfurthii* pulp oil, arranged in the ascending order based on their respective chemical formulae and retention times. For emphasis, the caprylic acid obtained the least carbon chain ratio (C8:0), whereas the lignoceric acid obtained the highest carbon chain ratio (C24:0). Moreover, the oleic acid (C18:0) obtained the highest fatty acid concentration of 74.56%, whereas the caprylic acid (C24:0), capric acid (C10:0), lauric acid (C12:0), and myristic acid (C14:0) were almost not detected in the oil. Georges, Olivier, and Simard^4^ reported high content of oleic acid (89.4%) and stearic acid (67.7–84%) of *C. schweinfurthii* pulp oil from the respective liquid and semi-solid parts of the oil, both well above those reported in this current study (oleic acid = 74.56%; stearic acid = 8.57%). The percentage oleic acid found in this study is related to total concentrations of the oleic acid (C18:1 n-9) and its isoforms (potentially C18:1 n−7 and C18:1 n−12, vaccenic acid and petroselinic acid, respectively). Besides the oleic acid being considered the topmost monounsaturated fatty acid in the human diet, the consumption of such (monounsaturated) fatty acid would help to increase the high-density lipoprotein (HDL), and decrease the low-density lipoprotein (LDL) cholesterol types^39,40^.

**Table 3:** Fatty acid profile of *C. schweinfurthii* pulp oil, based on compounds, retention time, concentration, carbon chain ratio, and chemical formulae.

Compound	Ret. time	Conc. (mg/mL)	Formula by carbon	Chemical formulae
Caprylic acid	8.902	0.00	C8:0	C_8_H_16_O_2_
Capric acid	11.056	0.00	C10:0	C_10_H_20_O_2_
Lauric acid	12.825	0.00	C12:0	C_12_H_24_O_2_
Myristic acid	14.491	0.00	C14:0	C_14_H_28_O_2_
Palmitic acid	16.040	9.23	C16:0	C_16_H_32_O_2_
Palmitoleic acid	16.602	0.42	C16:1	C_16_H_30_O_2_
Margaric acid^a^	17.365	0.00	C17:0	C_17_H_34_O_2_
Stearic acid	18.057	8.57	C18:0	C_18_H_36_O_2_
Oleic acid	18.937	74.56	C18:1	C_18_H_34_O_2_
Linoleic acid	19.522	4.46	C18:2	C_18_H_32_O_2_
Linolenic acid	20.654	0.90	C18:3	C_18_H_30_O_2_
Arachidic acid	21.904	0.61	C20:0	C_20_H_40_O_2_
Arachidonic acid	22.995	0.35	C20:4	C_20_H_32_O_2_
Behenic acid	24.034	0.38	C22:0	C_22_H_44_O_2_
Erucic acid	24.878	0.28	C22:1	C_22_H_42_O_2_
Lignoceric acid	25.615	0.18	C24:0	C_24_H_48_O_2_

^a^Margaric acid methyl ester (C17:0) served as an internal standard.

The generalized fatty acid content values of *C. schweinfurthii* pulp oil, previously reported by Maduelosi and Angaye^8^, included oleic (~ 36%), linoleic (~ 28%), palmitic (~ 26%), and stearic (~ 7%) acids, all of which appears not to be in agreement with those of this current study. A number of reasons could be responsible for such differences in fatty acid content values at this current study, for instance, the location of the cultivated *C. schweinfurthii* tree crop, harvest time, as well as the applied oil extraction method(s). Nonetheless, there are other reported fatty acid profiles of resembling extracted seed oils that can be compared with those of this current work. For example, the oleic acid content of *C. albidum* seed oil (30.21%)^31^ and of *P. americana* seed oil (40.33%)^30^ fell below that of *C. schweinfurthii* pulp oil at this current study. Essentially, the presence of fatty acids could provide several physiological benefits to the human immune system^41^, which are crucial for body metabolism/energy production^42^. Given its high oleic acid concentration, the *C. schweinfurthii* pulp oil could help in managing the human dietary cholesterol by keeping the blood’s Low Density Lipoprotein (LDL) in check^43^.

### Carotenoids, sterols, and tocopherols of *C. schweinfurthii* pulp oil

In the context of plant products, HPLC serves as a quantitative testing method of (plant)chemical composition on one hand, and its quality assurance for increased productivity in the food industry, on the other hand. Typically and for analytical and preparative purposes, HPLC continues to serve as a powerful tool able to separate a large number of organic compounds, for instance, tocopherol and carotenoid^44–46^.The concentration of sterols, tocopherols and carotenoids of *C. schweinfurthii* pulp oil is shown in Table 4. Clearly, some essential phytonutrients (sterols, tocopherols and carotenoids) were detected as quantified by HPLC spectroscopic technique. Quantitatively, while the concentration trend of sterols were cholecalciferol (32.809 µg/100 mL) > campesterol (31.313 µg/100 mL) > ergocalciferol (21.678 µg/100 mL) > ergosterol (13.503 µg/100 mL) > sitosterol (0.690 µg/100 ml), and those of tocopherols were: $\alpha$-tocopherol (31.834 µg/100 mL) >  γ-tocopherol (24.319 µg/100 mL) > β-tocopherol (17.826 µg/100 mL) > δ-tocotrienol (0.524 µg/100 mL), those of carotenoids were: β-carotene (37.951 µg/100 mL) > γ-carotene (33.107 µg/100 mL) > α-carotene (12.420 µg/100 mL). These (above-mentioned) phytonutrients provide enormous physiological benefits, for instance, reducing the cholesterol metabolism^47^. The relative high carotene values suggests the *C. schweinfurthii* pulp oil of this current study an important nutritional resource. The more common phytosterols in oil containing foods especially those from plant sources can include sitosterol and campesterol^48^.

**Table 4:** Concentration of sterols, tocopherols and carotenoids of *C. schweinfurthii* pulp oil.

Sterols	(µg/100 ml)	Tocopherols	(µg/100 ml)	Carotenoids	(µg/100 ml)
Ergosterol	13.503	*α*-tocopherol	31.834	*α*-carotene	12.420
Cholecalciferol	32.809	*β*-tocopherol	17.826	*β*-carotene	37.951
Ergocalciferol	21.678	*γ*-tocopherol	24.319	*γ*-carotene	33.107
Campesterol	31.313	*δ*-tocotrienol	0.524		
Sitosterol	0.690				

Moreover, there are a number of factors that can influence the degree by which the auto-oxidation process takes place, which can include fatty acid composition, light, metal ions, polyphenols, temperature, and tocopherols^34^. The concentration of campesterol of *C. schweinfurthii* pulp oil (31.313 µg/100 mL) at this current study fell below those of cold pressed coconut oil^48^, and together with sitosterol, competes well with other edible oils reported elsewhere^49,50^. This current result of *C. schweinfurthii* pulp oil appears to relate with another previous research where both campesterol and sitosterol appeared as the dominant phytosterols^51^. Particularly, the presence of campesterol and sitosterol in *C. schweinfurthii* pulp oil of this current work was actualized, owing to the Soxhlet extraction that employed organic solvent with a moderate temperature^22^. As a plant sterol, campesterol possesses the anticarcinogenic capacity, which could lower the cholesterol^52^. Considering the apparent synergistic stimulatory effect of sitosterol on the immune system, it would be desirable to consume sufficient (sitosterol-containing) unprocessed/unrefined plant foods^53^.

Generally, plant as well as vegetable oils comprise a number of bioactive constituents, which include tocol-related compounds, e.g., tocopherols, tocotrienols, etc. Specifically, tocopherols are well known vitamin E compounds that possess saturated phytyl chain, whereby the α-, β-, γ-, δ-types are differentiated based on the location and number of methyl constituents within the chroma ring^54^. As demonstrated in Table 4, the total concentration of tocopherols in *C. schweinfurthii* pulp oil was ~ 73 mg/100 g, which comprised α-, β-, and γ-tocopherol, as well as δ-tocotrienol ,detected at varying concentrations, which appeared above those that Franke et al.^45^ reported for rapeseed oil (~ 68.0 mg/100 g). Further, the relative abundance of tocopherol in *C. schweinfurthii* pulp oil makes it a reliable source of natural antioxidant. Moreover, previously reported vegetable oils of corn and soybean seed^45,55^, as well as palm oil^56^ signals that variations in the amounts/concentrations of carotenoids and tocopherol should be expected in oil seeds. Additionally, the carotenoid content of the oils of plant seed would supplement their antioxidant potential/value^55^. A high concentration of α- and γ- tocopherol has been reported in canola, sunflower and corn oil^57^. The tocopherol, although needful in tiny concentrations to maintain good human health^45^, which being present in the *C. schweinfurthii* pulp oil of this current study would suggest it as promising in scavenging the free radicals^20^. Besides, processing methods would contribute to considerably reduce the quantities of carotenoids usually detected in the raw nature of plant oil^56,58^.

## Conclusions

The proximate, lipid oxidation, fatty acid profile, carotenoids, sterols, and tocopherols of Soxhlet-extracted pulp oil of *C. schweinfurthii* fruit specific to South-east of Nigeria has been successfully investigated. For emphasis, the processing of the fruit sample to pulp oil involved, among others, oven-drying, and grinding, prior to the Soxhlet extraction, the latter of which employed n-hexane as the solvent, and resulted in promising pulp oil yield. Further, the proximate components, lipid peroxidation and fatty acid features/profile, together with the concentrations of sterols, tocopherols and carotenoids have cumulatively helped to demonstrate the biochemical importance of the *C. schweinfurthii* pulp oil. Potentially, the instances of high quantities of oleic acid, carotene, as well as tocopherol makes the pulp oil of this study nutritionally important, and biochemically competitive with strong industrial promise. Given the findings of the current work, it would be useful for future studies to investigate the group of flavonoids, which includes anthocyanins and condensed tannins, given that the content of these two groups could help to further unravel the richness of the pulp oil. In addition, future studies could also investigate the changes in lipid peroxidation of the *C. schweinfurthii* pulp oil when subject to varied storage conditions, because such new data would provide additional relevant information, not only about its biochemical/nutritional status but also its industrial potential.

## Data Availability

The datasets generated during and/or analyzed during the current study are available from the corresponding authors E.C.O., and C.O.R.O. on reasonable request.
